# Plating of a Single Bone Is Promising for the Treatment of Both-Bone Forearm Fractures in Children

**DOI:** 10.3390/life16060978

**Published:** 2026-06-10

**Authors:** Shou En Cheng, Kai Xuan Lim, Shang-Ming Lin, Ching-Ting Liang, Tsung-Yu Lan

**Affiliations:** 1 Department of Orthopedic Surgery, Far Eastern Memorial Hospital, New Taipei City 220, Taiwan; samuel830125@gmail.com (S.E.C.); kaixuanlim89@gmail.com (K.X.L.); ting905487@gmail.com (C.-T.L.); 2 Department of Materials and Textiles, Asia Eastern University of Science and Technology, New Taipei City 220, Taiwan; fc013@mail.aeust.edu.tw

**Keywords:** pediatric forearm both-bone fracture, plating, single-bone fixation

## Abstract

Background: Forearm fractures involving both bones are common orthopedic injuries. Children have a higher tolerance for greater displacement and angulation owing to the remodeling potential. The optimal fixation method for managing pediatric forearm fractures has not been definitively established. This study evaluated the safety and efficacy of a stepwise surgical algorithm, wherein single-bone plating was attempted first, and both-bone fixation was strictly reserved for cases demonstrating persistent intraoperative instability. Methods: In this retrospective analysis, we evaluated 48 skeletally immature children with both-bone forearm fractures managed via our stepwise protocol. Initially, single-bone plating was performed. Dynamic manual stress testing was then applied under fluoroscopy. If the unplated bone exhibited rotational instability, residual angulation >15°, or translation >50%, the procedure was converted to both-bone plating (Group B, n = 16). Patients who achieved stable alignment without requiring a second plate formed Group A (n = 32). Results: Both groups achieved 100% union. Postoperative angulations of the radius on the anteroposterior view were 1.91 ± 2.73° in Group A and 0.88 ± 1.96° in Group B; meanwhile, the lateral angulation of the radius in Groups A and B was 1.88 ± 3.56° and 0.00 ± 0.00°, respectively. The anteroposterior angulation of the ulna was 2.31 ± 3.60° in Group A and 2.19 ± 4.00° in Group B, whereas the lateral angulation of the ulna was 2.81 ± 3.74° in Group A and 1.75 ± 3.47° in Group B. Only the lateral angulation of the radius showed a significant difference (*p* = 0.0418). In the subgroup analysis, minor differences in ulna angulation on the anteroposterior view reached statistical significance in the older cohort (*p* = 0.027) and in the distal-third fracture group (*p* = 0.001). No differences in bone healing or functional outcomes were observed, and complication rates were similar. Conclusion: Our stepwise surgical algorithm appears to be a safe and effective approach. By adhering to this protocol, 66.7% of patients were successfully spared the morbidity of a second incision, while all patients achieved solid union and excellent functional outcomes. However, further high-quality studies are essential to establish comprehensive protocols for intraoperative stability assessment and postoperative care.

## 1. Background

Pediatric both-bone forearm fractures are a common orthopedic injury, accounting for approximately 3–6% of all fractures in children [1]. The immature skeletal structures in children allow for varying degrees of angulation and displacement, which are influenced by the child’s age. Initial management typically involves non-operative approaches, such as closed reduction and casting. Nevertheless, instances of conservative treatment failure have been documented [2]. Several indices, including the cast index, padding index, Canterbury index, gap index, and 3-point index, have been developed to predict the risk of redisplacement [3,4]. Besides indices evaluating the quality of casting, host factors such as obesity, age, and fracture patterns contribute to the loss of reduction [5,6,7]. In response to the failure of conservative treatment, there is a growing preference for surgical intervention, particularly for fractures that do not achieve acceptable alignment parameters after closed reduction and manipulation. Intramedullary nailing has been a well-established fixation technique for decades, whereas plating has recently emerged as a viable alternative [8]. The concept of single-bone plating for pediatric both-bone forearm fractures has evolved significantly over the past two decades. Early seminal studies, such as the work by Bhaskar and Roberts [9], demonstrate that plating a single bone is often clinically adequate and effectively minimizes surgical trauma. Building upon this foundation, recent high-quality literature has further validated this approach. A randomized controlled trial by Khaled et al. [10] provided strong Level I evidence, confirming that single-bone plating yields comparable functional and radiographic outcomes to both-bone plating, while significantly reducing operative time and soft tissue dissection. To bridge the current gaps in intraoperative decision-making, our study introduces a practical, stepwise surgical algorithm. Rather than comparing single- and both-bone plating as competing alternatives for identical fracture severities, we aimed to evaluate the safety and clinical outcomes of a protocol where single-bone plating is attempted first, and both-bone fixation is strictly reserved for cases demonstrating residual instability under dynamic manual stress.

## 2. Methods

### 2.1. Patients

In this retrospective analysis, we collected data from a consecutive series of skeletally immature children who underwent surgical intervention for radioulnar diaphyseal displaced fractures at our hospital between April 2013 and June 2018. Demographic information and surgical details were extracted from patients’ medical records, and radiographic data were thoroughly reviewed. Diaphyseal fractures were defined as those occurring between the distal and proximal metaphyses. Patients with open fractures, Monteggia fractures, Galeazzi fractures, multiple fractures, or inadequate radiographic data were excluded.

### 2.2. Surgical Procedures

**Figure 1:**
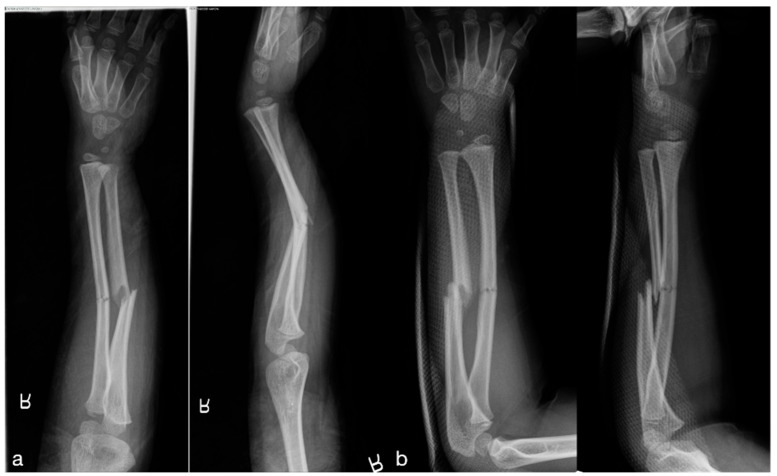
(**a**) Forearm fracture before closed reduction. (**b**) Residual angulation and translation of the ulna after manipulation.

A single pediatric orthopedic surgeon, who is also the corresponding author, conducted all the surgical procedures. The fractures were manually manipulated and reduced under general anesthesia, followed by fluoroscopic confirmation (Figure 1). Open reduction and internal fixation were performed only if the angulation of either the radius or ulna exceeded 15°. During surgery, patients were placed in the supine position, and a tourniquet was applied for hemostasis. The modified Henry approach was used for radial fractures, whereas the standard ulnar approach was employed for ulnar fractures. Fixation was achieved using 2.4 mm locking plates (DePuy Synthes, West Chester, PA, USA), ensuring secure attachment with a minimum of four cortices on each side of the fracture.

**Figure 2:**
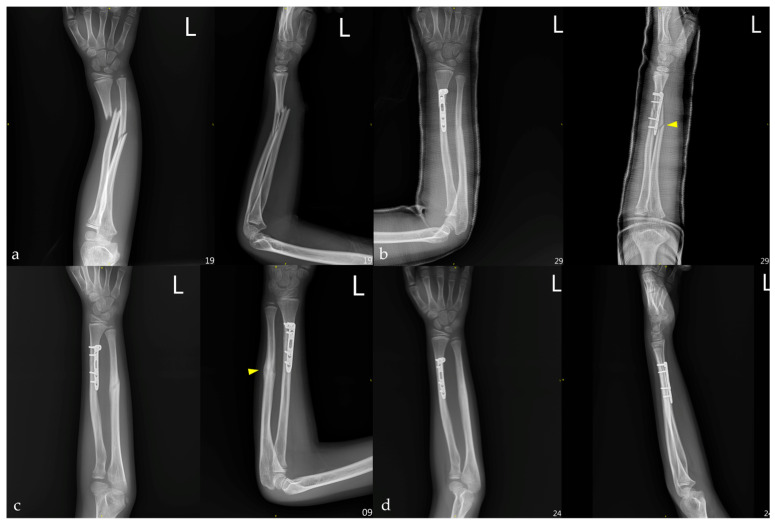
Both-bone forearm fracture fixed with a single plating technique. (**a**). Left both-bone forearm fracture with angulation of the radius and ulna. (**b**). Two weeks after the fixation, a residual 10° angulation of the unfixed ulna is noted on the lateral view. (**c**). Seven weeks after fixation, hard callus formation is noted around both fractures; the angulation of the ulna has decreased. (**d**). Ten weeks after fixation, a solid union of the both-bone fracture is noted; no residual angulation is found.

In our stepwise protocol, fractures were classified by location to guide initial single-bone stabilization: radial plating for distal-third fractures, and ulnar plating for middle- or proximal-third fractures (Figure 2). This novel decision-making algorithm builds on established anatomical principles [9,11,12]. The ulna was prioritized proximally to avoid posterior interosseous nerve (PIN) injury [11] and re-establish the stable rotational axis [12]. Distally, the radius was prioritized as the primary deforming bone, since restoring the radial bow is critical for full pronation and supination [9].

Following initial fixation, the unplated bone was dynamically evaluated under fluoroscopy using manual stress. Patients achieving acceptable alignment—defined as <15° of anteroposterior and lateral angulation [11] with ≤50% translation—were successfully managed with single-bone fixation (Group A).

**Figure 3:**
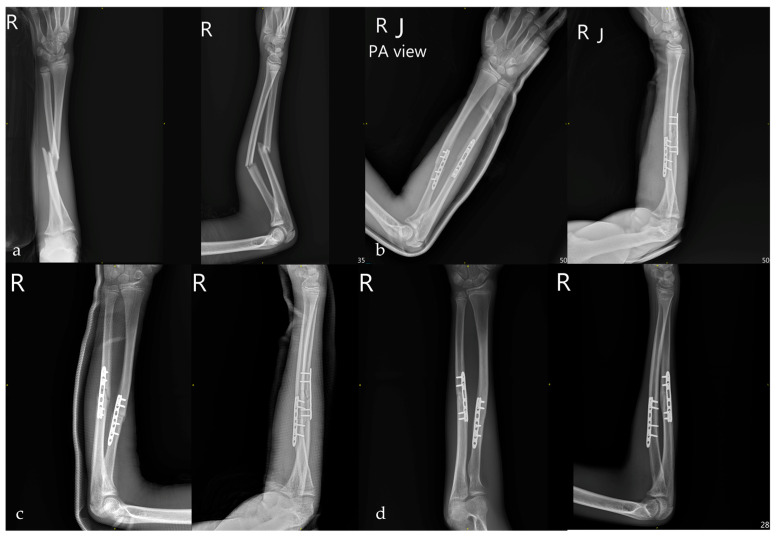
Both-bone forearm fracture fixed with the double plating technique. (**a**). Right both-bone forearm fracture with angulation of the radius and ulna. (**b**). Postoperative image after fixation. Fixation of both bones achieved anatomical reduction. (**c**). Four weeks after fixation, hard callus formation is noted around both fractures. (**d**). Twelve weeks after fixation, a solid union of the both-bone fracture is noted.

Conversion to both-bone plating (Group B, Figure 3) was strictly indicated for unacceptable angulation, excessive translation, or persistent rotational instability. To minimize subjectivity and account for the high variance of static rotational landmarks [13], rotational instability was dynamically standardized. The surgeon verified the absence of any mechanical block to full passive rotation. Concurrently, fluoroscopic profiles of the proximal radius (bicipital tuberosity) and distal ulna (ulnar styloid) were tracked during movement. Restricted motion, palpable crepitus, or dynamic narrowing of the interosseous space indicated unacceptable radioulnar impingement, mandating immediate conversion to both-bone plating.

### 2.3. Rehabilitation and Follow-Up

Postoperatively, all patients underwent long-arm splinting for 4 weeks. Postoperative radiographs were obtained on the day of the procedure and monthly thereafter until union was achieved. Union was defined as the radiological presence of a bridging callus at three out of four cortices on the AP and lateral views. In our cohort, plate removal was not routinely required. The decision to remove the implant was made through thorough discussions with patients and their families. When plate removal was indicated, the procedure was generally performed 6 months to 2 years following internal fixation.

### 2.4. Radiographic Assessment

All preoperative and postoperative radiographs were retrospectively reviewed. To ensure the reliability of the measurements and mitigate potential single-observer bias, radiographic angulations were independently re-evaluated by two observers (blinded to the clinical outcomes). The inter-observer reliability was determined using the Intraclass Correlation Coefficient (ICC).

Furthermore, to assess the initial severity of the fractures and ensure baseline comparability between the two groups, preoperative translational displacement was evaluated. To avoid magnification errors inherent in plain radiographs lacking calibration markers, translation was categorized based on the degree of cortical contact on the maximum displaced view (either anteroposterior or lateral). Fractures were classified into three standardized grades: Grade 0 (anatomic reduction/no displacement), Grade 1 (partial translation with remaining cortical contact), and Grade 2 (total translation, representing complete loss of cortical contact or bayonet apposition).

### 2.5. Statistical Analysis

Continuous variables are presented as mean ± standard deviation or median ± interquartile range, whereas categorical variables are expressed as numbers and percentages (%). Continuous variables were first evaluated for normality using the Shapiro–Wilk test. Variables that met the assumption of normality, such as tourniquet time and union time (*p* > 0.05), were analyzed using the independent Student’s *t*-test and presented as mean ± standard deviation. Conversely, postoperative radiographic angulation measurements demonstrated a non-normal, skewed distribution (*p* < 0.05) due to the high frequency of near-anatomic reductions (0° angulation). Consequently, comparisons of angulation between the single-bone and both-bone groups were performed using the non-parametric Mann–Whitney U test. Radiographic parameter comparisons within the same group were conducted using repeated measures ANOVA. Pearson’s chi-square test or Fisher’s exact test was used to analyze categorical data, depending on the sample size. Statistical significance was set at *p* < 0.05. Statistical analyses were performed using SPSS 20.0 (IBM Corp., Armonk, NY, USA).

## 3. Results

Of the 48 patients with radioulnar diaphyseal fractures included in this study, 32 underwent single-bone fixation, whereas 16 underwent fixation of both the radius and ulna. The mean patient ages were 10.5 ± 2.6 years in the single-bone fixation group and 11.5 ± 2.0 years in the both-bone fixation group. The mean follow-up period for the single-bone and both-bone fixation groups was 7.8 ± 3.3 and 7.5 ± 2.9 months, respectively (overall range, 2–17 months). Notably, no significant differences were observed in other demographic data, including sex, fracture subtype, or duration of follow-up (Table 1). Preoperative translation was assessed to compare the initial fracture severity between the two groups. There was no statistically significant difference in the distribution of initial translation grades between the single-bone (n = 32) and both-bone (n = 16) groups (*p* = 0.779). This indicates that the choice of single-bone fixation was not restricted to less severe fractures, confirming that the baseline radiographic severity was comparable between the cohorts.

Regarding radiological outcomes, the preoperative angulations of the radius in the single-bone fixation group were 11.5° and 19.6° in the AP and lateral views, respectively. The ulnar angulations were 10.0° and 20.0° on the AP and lateral views, respectively. In the both-bone fixation group, the preoperative angulations of the radius were both 15.1° on the AP and lateral views, respectively, whereas those of the ulna were 13.1° and 14.7°, respectively. Preoperative forearm deformities did not show a significant difference between the two fixation groups. The average postoperative angulations of the radius on the AP view were 1.91 ± 2.73° and 0.88 ± 1.96°, whereas those in the lateral view were 1.88 ± 3.56° and 0.00 ± 0.00°, for the single-bone and both-bone fixation groups, respectively. The effect sizes for these differences were small to moderate (Cohen’s *d* = 0.41 and 0.64, respectively). The postoperative angulations of the ulna on AP view were 2.31 ± 3.60° and 2.19 ± 4.00°, whereas those in the lateral view were 2.81 ± 3.74° and 1.75 ± 3.47° for the single-bone and both-bone fixation groups, respectively (Table 2 and Table 3). The corresponding effect sizes were small (Cohen’s *d* = 0.03 and 0.29). The angulations of the forearm were significantly corrected in both the single-bone and both-bone fixation groups, indicating successful intraoperative reduction. When comparing the two cohorts, only the lateral angulation of the radius showed a significant difference postoperatively (1.88 ± 3.56° vs. 0.00 ± 0.00°, *p* = 0.0418) (Table 4).

The inter-observer reliability for the radiographic angulation measurements was evaluated separately for the radius and ulna in both anteroposterior (AP) and lateral views. The ICCs demonstrated excellent agreement across all parameters: 0.973 (95% CI: 0.960–0.982) for the radius AP view, 0.977 (95% CI: 0.966–0.985) for the radius lateral view, 0.984 (95% CI: 0.976–0.989) for the ulna AP view, and 0.961 (95% CI: 0.942–0.974) for the ulna lateral view.

Since the ability for fracture remodeling varies with age, the acceptable degree of reduction should be stratified by both age and fracture location. To date, most studies have followed the guidelines established by Noonan and Price [14]. In line with this, we conducted a subgroup analysis based on age and fracture location (see Supplementary Tables S1–S4). Only minor differences in ulna angulation on the anteroposterior view reached statistical significance in the older cohort (0.06 ± 0.25 vs. 1.50 ± 2.46, *p* = 0.027) and in the distal-third fracture group (0.17 ± 0.65 vs. 2.33 ± 2.66, *p* = 0.001).

In terms of functionality, we categorized the postoperative functional outcomes based on the Price grading system [15]. Two patients who underwent single-bone fixation were categorized as having “good” functional outcomes, and the remaining patients, including 30 patients in Group A and 16 in Group B, presented “excellent” functional outcomes. The functional outcomes did not differ between the groups (Table 5).

Both groups achieved a 100% union rate. The single-bone fixation group had significantly shorter tourniquet times than the both-bone fixation group (32.0 ± 4.9 min vs. 69.3 ± 6.0 min). Additionally, the single-bone fixation group demonstrated a shorter time to union, although the difference between the two groups was not significant. Regarding complications, two cases (6.3%) of superficial skin infection were documented in the single-bone fixation group, whereas three cases (18.8%), including one case of superficial skin infection and two of wound dehiscence, were noted in the both-bone fixation group (Table 6). Other documented complications, including malunion, nonunion, refracture, and synostosis, were not observed in either group. Notably, no significant differences were observed in the complication rates between the two groups.

## 4. Discussion

The incidence of pediatric forearm both-bone fractures has increased over the past few decades, leading to a corresponding rise in surgical intervention for this condition [14,16]. Although children have a capacity for bone remodeling, which allows conservative treatments such as closed reduction and casting, surgical intervention is still necessary for cases with malreduction, suboptimal alignment (excessive angulation), and malunion. Conservative treatment has shown higher rates of reangulation and redisplacement than those of internal fixation [17]. A previous study established that risk factors contributing to the failure of conservative treatment include age older than 10 years, initial ulnar angulation exceeding 15°, and a proximal radius fracture [11].

As the mainstay of treating pediatric forearm fractures, the results of intramedullary nailing have been well established [18,19,20,21,22]. Intramedullary nailing offers advantages, such as small incisions, short operative times and hospital stays, early postoperative mobility and easy implant removal. In contrast, open reduction with plating ensures precise correction of fracture angulation and malrotation, leading to rigid biomechanical stability. Despite these benefits, plating is associated with soft tissue dissection, periosteal stripping, and subsequent nonunion [23]. Previous systematic reviews have not revealed significant differences in functional outcomes and range of motion between intramedullary nailing and plating [18,24,25,26]. To mitigate the soft-tissue complications of traditional plating while preserving its rigid stability, we advocate for a single-bone fixation strategy. Our approach is strongly supported by the recent randomized controlled trial by Khaled et al. [10], which demonstrated that single-bone plating provides functional and radiographic outcomes comparable to those of both-bone fixation while significantly reducing surgical trauma.

To minimize soft tissue injury while attaining an acceptable reduction, a single-bone fixation strategy has emerged in the past decade [27]. Single-bone intramedullary fixation requires a shorter operative time with similar functional outcomes, including elbow and wrist range of motion, when compared to both-bone intramedullary fixation. However, the effectiveness of single-bone intramedullary fixation remains controversial. Although most studies focusing on single-bone intramedullary nailing have reported outcomes comparable to those of both-bone intramedullary nailing [19,20,28,29,30,31], other studies have shown an increased risk of fracture redisplacement and compromised functional outcomes [32,33]. To attain the balance between soft tissue preservation and sufficient stability, hybrid fixation has been proposed, which also has demonstrated good functional outcomes [34,35].

While clinical evidence regarding single-bone plating remains relatively sparse in the current literature [36], our study provides critical insights by evaluating a standardized, stepwise surgical protocol. Compared with both-bone plating, single-bone plating effectively reduces soft tissue dissection, tourniquet time, and length of hospital stay. In our series, both surgical interventions resulted in a solid fracture union. The time to union did not differ between the two groups, and no reangulation was observed in either group. In the subgroup analysis, radiographic outcomes between younger (≤10 years) and older (>10 years) patients did not reach statistical significance. Additionally, postoperative angulation of the forearm showed no clinical significance in relation to the location of the fractures. The results of the subgroup analysis suggest that single-bone fixation can achieve outcomes comparable to those of double-bone fixation, even in patients approaching skeletal maturity. Of note, these results must be interpreted cautiously. The absolute mean differences in angulation were minimal (less than 2 degrees). We believe that such microscopic radiographic differences are not clinically meaningful and do not translate to any discernible functional deficit. A potential criticism is selection bias, particularly the assumption that less invasive procedures (single-bone plating) are reserved exclusively for less severe fractures. However, our baseline evaluation of initial translational displacement refutes this assumption. Our data demonstrated that a substantial proportion of patients in the single-bone group (62.5%) presented with complete loss of cortical contact (bayonet apposition), which was statistically comparable to the both-bone group (66.7%). Crucially, as highlighted during the peer review process, initial radiographic severity does not reliably predict intraoperative dynamic instability. This finding implies that our algorithm does not arbitrarily filter patients based on preoperative radiographs. Instead, even fractures with severe initial displacement can be successfully and safely managed with single-bone fixation, provided that intraoperative dynamic manual stress testing confirms adequate rotational and angular stability after fixing the first bone.

Regarding postoperative complications, patients who underwent single-bone plating reported fewer complications, including wound dehiscence and superficial skin infections, than those reported by patients in the both-bone plating group. We believe that the lower complication rate, although not significant, resulted from less soft tissue dissection. The functional outcomes were promising in both cohorts, consistent with those of previous studies [9,10]. Additionally, apart from the favorable postoperative results achieved through single-bone plating, hospitalization costs were significantly lower. Moreover, smaller incisions contributed to improved cosmetic outcomes and positively influenced psychological development. While the debate over whether to initially fix the radius or ulna in single-bone plating remains historically unresolved [12], our location-based bone selection protocol provides a standardized solution. By strategically favoring the ulna for proximal and middle fractures and the radius for distal fractures, our approach successfully translates established anatomical and biomechanical principles into clinical practice. This tailored strategy not only ensures optimal biomechanical stability but also significantly minimizes surgical time and the extensive soft-tissue dissection—along with associated nerve risks—required for proximal radius exposure.

This study had some limitations, including its retrospective design and inclusion of a modest sample size, which could have potentially yielded underpowered results. To provide a more nuanced interpretation of our results beyond *p*-values, we calculated effect sizes (Cohen’s *d*) for the primary radiographic outcomes. The trivial to small effect sizes observed (typically d < 0.2) underscore that the magnitude of the difference between the two groups is minimal. While this does not constitute a formal proof of non-inferiority, it provides quantitative evidence that the final radiographic alignment achieved through our stepwise algorithm is highly consistent, regardless of whether a second plate was ultimately required. Another limitation is our reliance on the Price grading system, which demonstrated a ceiling effect in our functional assessment. Because of the retrospective nature of this study, detailed Patient-Reported Outcome Measures (PROMs) and precise degrees of range of motion were not routinely recorded. Despite the increased incidence of bilateral fractures of the pediatric forearm in recent decades, the proportion of patients requiring surgical fixation for unstable fractures remains relatively low. Additionally, the incidence of complications such as malunion, nonunion, and reangulation varied by age [37]. A prospective cohort study with a large sample size and a long follow-up period, incorporating validated PROMs (such as the QuickDASH score), should be conducted in the future to validate the findings of this paper. A large sample size would also enhance the credibility of subgroup analyses, especially age stratification accounting for gender differences, which was not performed in our study due to an insufficient sample size.

## 5. Conclusions

Our stepwise surgical algorithm—attempting single-bone plating first and converting to both-bone fixation only when objective intraoperative instability is present—may represent a safe and effective approach for treating pediatric both-bone forearm fractures. This protocol demonstrates favorable clinical outcomes while reducing operative time and hospital stay. However, considering the retrospective, single-center design and the small sample size of our study, further high-quality prospective studies are required to validate these findings, standardize intraoperative stability evaluations, and refine postoperative protocols.

## Figures and Tables

**Table 1:** Demographic data of the study population.

	Single-Bone Fixation (Group A) n = 32	Both-Bone Fixation (Group B) n = 16	*p*-Value
Age, years, mean ± SD	10.5 ± 2.6	11.5 ± 2.0	0.19 †
Sex, no., %			0.58 ‡
Female	6 (18.8)	2 (13)	
Male	26 (81.2)	14 (87)	
Subtype of fracture, no.			0.07 ‡
Distal third	23	6	
Middle third	8	9	
Proximal third	1	1	
Time of follow-up, month, mean ± SD	7.8 ± 3.3	7.5 ± 2.9	0.91 †
Translation			0.78 ‡
Grade 0	1	0	
Grade 1	11	5	
Grade 2	20	10	

*p*-value < 0.05 is considered significant; † Mann–Whitney U test; ‡ Pearson’s chi-squared test; SD, standard deviation.

**Table 2:** Radiographic alignment of the single-bone fixation group (Group A).

	Preoperative (Degree)	Postoperative (Degree)	Follow-Up (Degree)	*p*-Value
Radius, AP				
Average	11.53 ± 10.74	1.91 ± 2.73	0.34 ± 1.36	<0.001 *
Median (IQR)	10 (12)	0 (3.25)	0 (0)	
Range	0–56	0–10	0–6	
Radius, Lat				
Average	19.63 ± 12.06	1.88 ± 3.56	0.59 ± 1.43	<0.001 *
Median (IQR)	19.5 (14.25)	0 (3.25)	0 (0)	
Range	0–59	0–12	0–5	
Ulna, AP				
Average	10.06 ± 10.17	2.31 ± 3.60	0.41 ± 1.21	<0.001 *
Median (IQR)	8.5 (17.25)	0 (3.25)	0 (0)	
Range	0–35	0–14	0–5	
Ulna, Lat				
Average	20.03 ± 12.79	2.81 ± 3.74	0.38 ± 0.98	<0.001 *
Median (IQR)	20 (19.25)	0 (4.5)	0 (0)	
Range	0–45	0–13	0–4	

*p*-value < 0.05 is considered significant; * Repeated measured ANOVA; AP, anteroposterior; Lat, lateral; IQR, interquartile range.

**Table 3:** Radiographic alignment of both-bone fixation (Group B).

	Preoperative (Degree)	Postoperative (Degree)	Follow-Up (Degree)	*p*-Value
Radius, AP				
Average	15.13 ± 11.46	0.88 ± 1.96	0.25 ± 1.00	<0.001 *
Median (IQR)	13.5 (18.75)	0 (0)	0 (0)	
Range	0–33	0–6	0–4	
Radius, Lat				
Average	15.13 ± 11.46	0.00 ± 0.00	0.00 ± 0.00	<0.001 *
Median (IQR)	18.5 (11.75)	0 (0)	0 (0)	
Range	0–40	0–2	0	
Ulna, AP				
Average	13.06 ± 13.57	2.19 ± 4.00	1.19 ± 2.17	<0.001 *
Median (IQR)	9 (16.5)	0 (2.5)	0 (1)	
Range	0–45	0–13	0–6	
Ulna, Lat				
Average	14.69 ± 8.86	1.75 ± 3.47	0.00 ± 0.00	<0.001 *
Median (IQR)	13 (13)	0 (1.25)	0 (0)	
Range	0–28	0–12	0	

*p*-value < 0.05 is considered significant; * Repeated measured ANOVA; AP, anteroposterior; Lat, lateral; IQR, interquartile range.

**Table 4:** Comparison of radiographic outcomes.

	Single-Bone Fixation (Group A, Degree) n = 32	Both-Bone Fixation (Group B, Degree) n = 16	*p*-Value	Cohen’s *d*
Radius AP				
Pre-op Post-op (Day 0) Post-op (Union)	11.53 ± 10.74 1.91 ± 2.73 0.34 ± 1.36	15.13 ± 11.46 0.88 ± 1.96 0.25 ± 1.00	0.29 † 0.18 † 0.81 †	0.41
Radius Lat				
Pre-op Post-op (Day 0) Post-op (Union)	19.63 ± 12.06 1.88 ± 3.56 0.59 ± 1.43	15.13 ± 11.46 0.00 ± 0.00 0.00 ± 0.00	0.76 † 0.04 † 0.11 †	0.64
Ulna AP				
Pre-op Post-op (Day 0) Post-op (Union)	10.06 ± 10.17 2.31 ± 3.60 0.41 ± 1.21	13.06 ± 13.57 2.19 ± 4.00 1.19 ± 2.17	0.39 † 0.91 † 0.11 †	0.03
Ulna Lat				
Pre-op Post-op (Day 0) Post-op (Union)	20.03 ± 12.79 2.81 ± 3.74 0.38 ± 0.98	14.69 ± 8.86 1.75 ± 3.47 0.00 ± 0.00	0.14 † 0.35 † 0.13 †	0.29

*p*-value < 0.05 is considered significant; † Mann–Whitney U test; AP, anteroposterior; Lat, lateral; Pre-op, preoperative; post-op, postoperative; Effect sizes (Cohen’s *d*) were interpreted as trivial (<0.2), small (0.2–0.49), moderate (0.5–0.79), or large (≥0.8).

**Table 5:** Comparison of functional outcomes.

	Single-Bone Fixation (Group A) n = 32	Both-Bone Fixation (Group B) n = 16	*p*-Value
Price grading			0.80 ‡
Excellent (≤10° loss of rotation)	30 (93.75%)	16 (100%)	
Good (11 to 30° loss of rotation)	2 (6.25%)	0	
Fair (≥30° loss of rotation) Poor	0 0	0 0	

*p*-value < 0.05 is considered significant; ‡ Fisher’s exact test.

**Table 6:** Surgical outcomes.

	Single-Bone Fixation (Group A) n = 32	Both-Bone Fixation (Group B) n = 16	*p*-Value	Cohen’s *d*
Union rate, %	100	100	1.00 ‡	
Time to union, day, mean ± SD	51.7 ± 15.8	52.3 ± 18.8	0.79 †	0.04
Tourniquet time, minutes, mean ± SD	32.0 ± 4.9	69.3 ± 6.0	<0.0001 †	7.06
Cost, $	2011.7 ± 143.3	3324.95 ± 140.7	0.0008 †	
Complication, no, %			0.1814 ‡	
Wound dehiscence	0	2 (12.5)		
Superficial skin infection	2 (6.3%)	1 (6.3)		

*p*-value < 0.05 is considered significant; † Independent Student’s *t* test; ‡ Fisher’s exact test; $ = US dollar, with currency rate $1 = 31 Taiwan dollars. SD, standard deviation.

## Data Availability

All data generated or analyzed during this study are included in this published article.

## Supplementary Materials

The following supporting information can be downloaded at: https://www.mdpi.com/article/10.3390/life16060978/s1, Table S1: Subgroup analysis of radiographic outcomes (≤10 y); Table S2: Subgroup analysis of radiographic outcomes (>10 y); Table S3: Subgroup analysis of radiographic outcomes (distal 3rd fractures); Table S4: Subgroup analysis of radiographic outcomes (middle/proximal 3rd fractures).

## Abbreviations

The following abbreviations are used in this manuscript:

AP	anteroposterior
SD	standard deviation
IQR	interquartile range
