# Supplementary material for: Plating of a Single Bone Is Promising for the Treatment of Both-Bone Forearm Fractures in Children
Source: Life (Basel). 2026 Jun 10;16(6):978. doi: 10.3390/life16060978 (PMC13301182; doi:10.3390/life16060978)
Supplement: Supplementary file 1 [file life-16-00978-s001.zip › life-4285589-supplementary.pdf]

Supplementary Materials

Table S1. Subgroup analysis of radiographic outcomes (≤ 10 y).

	**Single-bone fixation (Group A, degree) n = 16**	**Both-bone fixation (Group B, degree) n = 6**	***P*-value**
Radius AP			
Pre-op	12.56±13.59	12.00±12.31	0.90†
Post-op (Day 0)	2.06±3.32	0.00±0.00	0.15†
Post-op (Union)	0.69±1.89	0.00±0.00	0.39†
Radius Lat			
Pre-op	20.75±15.60	20.00±9.14	0.91†
Post-op (Day 0)	2.06±3.32	0.00 ± 0.00	0.15†
Post-op (Union)	0.44±1.21	0.00 ± 0.00	0.39†
Ulna AP			
Pre-op	10.56±10.42	9.17±12.45	0.79†
Post-op (Day 0)	3.13±4.60	0.00±0.00	0.12†
Post-op (Union)	0.75±1.65	0.67±1.63	0.92†
Ulna Lat			
Pre-op	21.38±14.38	13.83±9.06	0.25†
Post-op (Day 0)	2.75±4.14	2.67±2.94	0.96†
Post-op (Union)	0.19±0.75	0.00 ± 0.00	0.55†

P-value <0.05 is considered significant

†Mann–Whitney U test

AP, anteroposterior; Lat, lateral; Pre-op, preoperative; post-op, postoperative

Table S2. Subgroup analysis of radiographic outcomes (> 10 y).

	**Single-bone fixation (Group A, degree) n = 16**	**Both-bone fixation (Group B, degree) n = 10**	***P*-value**
Radius AP			
Pre-op	10.50±7.16	17.00±11.15	0.08†
Post-op (Day 0)	1.75±2.08	1.40±2.37	0.70†
Post-op (Union)	0.00±0.00	0.40±1.26	0.21†
Radius Lat			
Pre-op	18.50±7.38	19.00±9.59	0.88†
Post-op (Day 0)	2.00±4.13	0.20±0.63	0.19†
Post-op (Union)	0.75±1.65	0.00 ± 0.00	0.17†
Ulna AP			
Pre-op	9.56±10.22	15.40±14.30	0.24†
Post-op (Day 0)	1.50±2.03	3.50±4.65	0.14†
Post-op (Union)	0.06±0.25	1.50±2.46	0.03†
Ulna Lat			

Pre-op	18.69±11.29	15.20±9.19	0.42†
Post-op (Day 0)	2.88±3.42	1.20±3.79	0.26†
Post-op (Union)	0.56±1.15	0.00 ± 0.00	0.14†

P-value <0.05 is considered significant

†Mann–Whitney U test

AP, anteroposterior; Lat, lateral; Pre-op, preoperative; post-op, postoperative

Table S3. Subgroup analysis of radiographic outcomes (distal 3rd fractures).

	**Single-bone fixation (Group A, degree) n = 23**	**Both-bone fixation (Group B, degree) n = 6**	***P*-value**
Radius AP			
Pre-op	8.65±6.12	15.17±11.20	0.06†
Post-op (Day 0)	2.13±2.51	1.00±2.45	0.33†
Post-op (Union)	0.22±1.04	0.00±0.00	0.62†
Radius Lat			
Pre-op	17.65±10.41	19.00±5.97	0.78†
Post-op (Day 0)	1.78±3.46	0.00 ± 0.00	0.22†
Post-op (Union)	0.70±1.58	0.00 ± 0.00	0.30†
Ulna AP			
Pre-op	9.22±8.91	10.33±7.26	0.24†
Post-op (Day 0)	2.13±2.62	4.17±5.38	0.19†
Post-op (Union)	0.17±0.65	2.33±2.66	0.0011†
Ulna Lat			
Pre-op	18.83±12.76	13.00±9.84	0.31†
Post-op (Day 0)	3.65±3.95	3.83±4.83	0.92†
Post-op (Union)	0.52±1.12	0.00 ± 0.00	0.27†

P-value <0.05 is considered significant

†Mann–Whitney U test

AP, anteroposterior; Lat, lateral; Pre-op, preoperative; post-op, postoperative

Table S4. Subgroup analysis of radiographic outcomes (middle/proximal 3rd fractures).

	**Single-bone fixation (Group A, degree) n = 9**	**Both-bone fixation (Group B, degree) n = 10**	***P*-value**
Radius AP			
Pre-op	21.25±15.45	15.67±12.82	0.43†
Post-op (Day 0)	1.50±3.51	0.89±1.83	0.65†
Post-op (Union)	0.75±2.12	0.44±1.33	0.72†
Radius Lat			
Pre-op	24.63±16.07	19.89±11.53	0.49†
Post-op (Day 0)	0.88±1.64	0.22±0.67	0.29†
Post-op (Union)	0.38±1.06	0.00 ± 0.00	0.30†
Ulna AP			

Pre-op	13.75±13.27	16.33±16.53	0.73†
Post-op (Day 0)	3.13±5.84	1.11±2.67	0.37†
Post-op (Union)	1.13±2.10	0.56±1.67	0.54†
Ulna Lat			
Pre-op	22.88±14.00	16.11±9.02	0.25†
Post-op (Day 0)	0.75±2.12	0.56±1.67	0.84†
Post-op (Union)	0.00±0.00	0.00 ± 0.00	1.00†

P-value <0.05 is considered significant

†Mann–Whitney U test

AP, anteroposterior; Lat, lateral; Pre-op, preoperative; post-op, postoperative
